# Health and Exercise-Related Medical Issues among 1,212 Ultramarathon Runners: Baseline Findings from the Ultrarunners Longitudinal TRAcking (ULTRA) Study

**DOI:** 10.1371/journal.pone.0083867

**Published:** 2014-01-08

**Authors:** Martin D. Hoffman, Eswar Krishnan

**Affiliations:** 1 Department of Physical Medicine & Rehabilitation, Department of Veterans Affairs, Northern California Health Care System, and University of California Davis Medical Center, Sacramento, California, United States of America; 2 Department of Medicine, Stanford University School of Medicine, Palo Alto, California, United States of America; Universidad Europea de Madrid, Spain

## Abstract

Regular exercise is associated with substantial health benefits; however, little is known about the health impact of extreme levels of exercise. This study examined the prevalence of chronic diseases, health-care utilization, and risk factors for exercise-related injuries among ultramarathon runners. Retrospective, self-reported enrollment data from an ongoing longitudinal observational study of 1,212 active ultramarathon runners were analyzed. The most prevalent chronic medical conditions were allergies/hay fever (25.1%) and exercise-induced asthma (13.0%), but there was a low prevalence of serious medical issues including cancers (4.5%), coronary artery disease (0.7%), seizure disorders (0.7%), diabetes (0.7%), and human immunodeficiency virus (HIV) infection (0.2%). In the year preceding enrollment, most (64.6%) reported an exercise-related injury that resulted in lost training days (median of 14 days), but little nonattendance of work or school due to illness, injury, or exercise-related medical conditions (medians of 0 days for each). The knee was the most common area of exercise-related injury. Prior year incidence of stress fractures was 5.5% with most (44.5%) involving the foot. Ultramarathon runners who sustained exercise-related injuries were younger (p<0.001) and less experienced (p<0.01) than those without injury. Stress fractures were more common (p<0.01) among women than men. We conclude that, compared with the general population, ultramarathon runners appear healthier and report fewer missed work or school days due to illness or injury. Ultramarathon runners have a higher prevalence of asthma and allergies than the general population, and the prevalence of serious medical issues was nontrivial and should be recognized by those providing medical care to these individuals. Ultramarathon runners, compared with shorter distance runners, have a similar annual incidence of exercise-related injuries but higher proportion of stress fractures involving the foot, and it is the younger and less experienced ultramarathoners who appear most at risk for injury.

## Introduction

Participation in ultramarathons (i.e. foot races longer than the standard 42.2-km marathon distance) has increased exponentially over the past few years [1], [2]. With the expansion of participation in these events comes a need for knowledge about the health impact of training and participation, the medical needs of the participants, and the types of medical issues that might present during these events. A limited amount of information has been published on the medical needs of ultramarathon runners during events [3]–[11], but very little is known about the acute and chronic health issues of ultramarathon runners [5], [12].

Prior work examining health issues of ultramarathon runners has focused on entries in two 161-km ultramarathons in the United States [5], [12]. This work found that over half of these runners had suffered a running-related injury during the prior year that was severe enough to interfere with training [5], but that running injuries accounted for an average of only 0.7 days of work or school loss the prior year [12]. Overall, this group reported an average of only 2.8 days of missed work or school in the previous year from any injury or illness [12]; this is considerably lower than the average nonattendance at work among employed Americans of the same age range. It is not surprising that ultramarathon runners have good general health but commonly experience running related injuries. However, no prior study has examined a large group of ultramarathon runners to assess prevalence of chronic medical conditions and how these individuals make use of the medical system. Such information is valuable in understanding potential benefits and risks from levels of exercise beyond the moderate amounts known to have health benefits.

The Ultrarunners Longitudinal TRAcking (ULTRA) Study is a longitudinal health study of ultramarathon runners. The primary intent of the study is to assess potential health consequences related to the high levels of exercise performed by these individuals, but analysis of baseline enrollment information offers insight into the extent of chronic medical conditions in this group, their level of use of the medical care system, and the type of exercise-related injuries they sustain. Here we present the baseline medical information on this large cohort of ultramarathon runners.

## Methods

Enrollment in the ULTRA Study began in November, 2011 and was accomplished through direct electronic mailing to over 3,000 ultramarathon runners, postings on various ultramarathon-related web sites and blogs, advertisements in magazines related to ultramarathon running, and distribution of flyers at a number of the largest ultramarathons in the United States. Participation in the ULTRA Study required that the individual had completed at least one ultramarathon of 50 km or longer at some point in their life. The present analysis is from the initial 12-month enrollment period of retrospective data collection, and considers only those who were “active ultramarathoners” defined as those who had completed an ultramarathon within the 12 months prior to enrollment or who planned to complete an ultramarathon during 2010 or 2011 and who stated that they were regularly running and intended to run ultramarathons again. The study was approved by Institutional Review Boards of the VA Northern California Health Care System and Stanford University, and each subject voluntarily provided informed consent through electronic means.

All participants completed a secure web-based questionnaire that included demographic questions, and questions related to medical and exercise history. Exercise-related injuries in the prior year and lifetime history of stress fractures were assessed. Exercise history included information about running and ultramarathon running experience, estimates of running distance, percentage of time spent running on concrete or asphalt, percentage of total exercise time spent running, percentage of time spent exercising at a high intensity, and whether or not resistance exercise had been performed. Sick time from illness and injury, and training days lost from exercise-related injury during the prior 12 months were also assessed.

Incomplete, obviously invalid (e.g., use of clearly erroneous name or address) and duplicate responses were removed from the data set. Each variable was examined for major outliers, and subjects were contacted to verify the accuracy of such data. Findings from the questionnaire that were specific to exercise habits [13] and to sociodemographics, lifestyle behaviors, and body mass index [14] are reported elsewhere.

Descriptive analyses were performed and are presented. Median values are provided for some variables as the data generally did not fit a normal distribution when visually inspected and examined with the D'Agostino and Pearson omnibus normality test. Since ultramarathon runners are known to have a high incidence of running injuries and stress fractures [5], comparisons of select characteristics between those who had and had not sustained an exercise-related injury and stress fracture in the prior 12 months were made. Since these variables did not pass normality testing with the D'Agostino and Pearson omnibus normality test, the Mann Whitney test was used for analysis of continuous data. The Fisher's exact test was used for analysis of categorical data. Statistical significance was set at p<0.05.

## Results

### Study Population

Study participants numbered 1,212; of these, 94.7% had completed an ultramarathon within the prior 12 months. Median age was 42.3 years (range 18–81 years) and 68.0% were men. Most (93.5%) reported ethnic origin as white, not of Hispanic origin. Most (88.0%) were living in the United States. Median running distance during the prior 12 months was reported to be 3,347 km (range 418–12,134 km).

### Health and Medical Care Utilization

The prevalence of various medical conditions reported by the ultramarathon runners is shown in Table 1. Most common were allergies/hay fever (25.1%) and exercise-induced asthma (13.0%). There was a low prevalence of serious medical issues, including cancers (4.5%), coronary artery disease (0.7%), seizure disorders (0.7%), diabetes (0.7%), and human immunodeficiency virus (HIV) infection (0.2%). The proportions of ultramarathon runners using medication for various medical conditions are shown in Table 1. Medication use was reported by 28.3% of the ultramarathon runners.

**Table 1:** Point prevalence of various medical conditions among 1,212 active ultramarathon runners and the proportion who were using relevant medications.

Medical Condition	Condition Prevalence (%)	Using Medication (%)
Vascular Diseases/Disorders		
Arrhythmias or irregular heart beats	7.6	0.6
Hypertension	7.6	3.3
Varicose veins	2.4	0
Thrombophlebitis	1.2	0.5
Coronary artery disease	0.7	0.4
Coronary valve disease	0.6	0
Raynaud's disease	0.5	0
Cardiac hypertrophy	0.4	0.1
Stroke or transient ischemic attack	0.2	0.1
Lower extremity claudication/peripheral vascular disease	0.2	0.1
Bradycardia	0.2	0
Conduction abnormality	0.2	0
Anemia	0.2	0.1
Other	0.8	0.1
Lung Diseases/Disorders		
Exercise-induced asthma	13.0	5.9
Asthma (other than exercise-induced)	10.7	4.7
Chronic bronchitis or cough	2.3	0.2
Spontaneous pneumothorax	0.2	0
Pulmonary embolism	0.2	0.1
Other	0.6	0
Cancers		
Basal cell	1.6	0
Melanoma	1.4	0.2
Thyroid cancer	0.2	0.2
Brain cancer	0.2	0
Squamous cell	0.2	0
Prostate cancer	0.2	0
Other	0.7	0.1
Musculoskeletal Diseases/Disorders		
Chronic low back pain or lumbar disc problems	9.7	0.3
Osteoarthritis	4.6	0.6
Chronic neck pain or cervical disc problems	2.6	0.2
Osteoporosis	1.9	0.7
Rheumatoid arthritis	0.8	0.2
Osteopenia	0.3	0.2
Scoliosis	0.2	0
Fibromyalgia syndrome	0.2	0.1
Gout	0.2	0
Other	0.6	0.1
Neurological and Psychological Issues		
Depression or bipolar disease	11.8	2.4
Anxiety	7.8	2.1
Seizure disorder/epilepsy	0.7	0.2
Attention deficit disorder	0.6	0.3
Multiple sclerosis	0.4	0.2
Brain injury	0.2	0.2
Restless leg syndrome	0.2	0
Other	0.3	0.1
Gastrointestinal Diseases/Disorders		
Hemorrhoids	12.9	0.7
Gastroesophageal reflux disease	6.7	2.3
Colitis or irritable bowel or colon	4.8	0.5
Peptic ulcer disease	3.2	0.4
Gall bladder disease/stones	2.1	0
Liver problems, hepatitis or cirrhosis	1.5	0
Diverticulitis	1.1	0
Celiac disease	0.3	0
Gastritis	0.2	0.2
Other	0.3	0.2
Other Medical Issues		
Allergies/hay fever	25.1	7.3
Frequent severe headaches or migraines	5.9	1.3
Sleeping difficulties or sleep apnea	5.8	0.8
Thyroid disease (hypothyroidism or hyperthyroidism)	5.1	3.4
Kidney disorders/stones	4.5	0.2
Weight problem/obesity	4.3	0.1
Hyperlipidemia or hypercholesterolemia	4.1	1.5
Alcoholism or drug abuse	3.4	0
Disordered eating/anorexia	2.9	0
Prostate enlargement (not cancer)	2.5	0.3
Cataracts	2.0	0.1
Significant hearing problems	1.9	0
Significant vision problem other than cataracts	1.2	0.1
Incontinence	1.0	0.1
Glaucoma	0.8	0.2
Diabetes	0.7	0.2
Skin disease	0.4	0.4
HIV disease	0.2	0.2
Endocrine disorder (other than diabetes)	0.2	0.1
Other	1.4	0.5

Conditions reported by at least two individuals are included in the table.

Table 2 shows that this group had very little time lost from work or school due to illness and injury during the prior 12 months. Only 14% of the time lost from work or school was due to an exercise-related injury. There was little use of the medical care system during the prior 12 months, but 64% of the outpatient medical visits were for exercise-related issues (Table 3). The percentages reporting 0, 1, 2–3, 4–9, and 10 or more outpatient visits, excluding dental and emergency room visits, were 27.7%, 18.8%, 27.2%, 17.6%, and 8.7%, respectively. The percentage of individuals reporting to have visited various types of health-care professionals in the prior 12 months is shown in Table 4. Most had visited a dentist or dental hygienist and a primary care provider.

**Table 2:** Lost time from work or school and from training due to illness and injury during the prior 12 months among 1,212 active ultramarathon runners.

Extent of Loss From Injury or Illness	Mean	Median	SD	Range
Missed work or school due to any illness (days)	1.5	0	3.3	0–50
Missed work or school due to any injury (days)	0.7	0	9.1	0–300
Missed work or school due to any exercise-related injury (days)	0.3	0	1.9	0–50
In bed more than a half day due to any illness or injury (days)	1.0	0	2.3	0–30
Missed training due to any exercise-related injury (days)	13.8	5	22.5	0–240

**Table 3:** Use of the medical care system during the prior 12 months among 1,212 active ultramarathon runners.

Medical Care	Mean	Median	SD	Range
Use of hospital emergency room (number of visits)	0.2	0	0.5	0–9
Hospitalization (number of nights)	0.1	0	0.7	0–10
Medical visits, excluding dental and emergency room (number)	3.2	2	5.8	0–100
Medical visits specific to an exercise-related issue (number)	2.1	1	5.2	0–95
Surgeries or other surgical procedures (number)	0.1	0	0.4	0–4

**Table 4:** Percentage of 1,212 active ultramarathon runners reporting to have visited various types of health care professionals in the prior 12 months.

Type of Health Care Professional	Percent
Dentist or dental hygienist	73.0
General practice clinician	68.6
Obstetrician/gynecologist	55.7^a^
Massage therapist	47.6
Optometrist or ophthalmologist	41.8
Medical specialist (excluding obstetrician/gynecologist, psychiatrist and ophthalmologist)	25.3
Chiropractor	24.0
Physical, occupational, speech or respiratory therapist, or audiologist	21.8
Podiatrist	12.5
Mental health professional	8.3
Acupuncturist	6.6

^a^ Percentage based upon number of women.

### Exercise-Related Injuries and Illnesses

A majority, 933 (77.0%), of individuals in the study group reported exercise-related injuries in the prior 12 months. A total of 1,900 injuries were reported. Exercise-related injuries that resulted in lost training of at least 1 day in the prior year were reported by 783 (64.6%). These injuries accounted for a median of 5 days of missed training when all 1,212 ultramarathon runners were considered (Table 2) and a median of 14 days among the 783 ultramarathon runners who reported an injury resulting in lost training days. The distribution and incidence of various types and/or locations of exercise-related injuries are shown in Table 5. The incidence was highest for knee issues (24.0%). Sixty-seven individuals reported 70 stress fractures, an annual stress fracture incidence of 5.5%.

**Table 5:** Number, distribution, and incidence of various exercise-related injuries in the prior 12 months among 1,212 active ultramarathon runners.

Injury Type and/or Location	n	Distribution (%)	Incidence (%)
Fractures not involving the extremities	12	0.6	1.0
Upper extremity injuries including fractures	17	0.9	1.4
Back injuries	150	7.9	12.4
Iliotibial band issue	191	10.1	15.8
Hip flexor strain	106	5.6	8.7
Hamstring strain	143	7.5	11.8
Stress fracture involving femur/hip	6	0.3	0.5
Other leg, pelvis or hip issues	45	2.4	3.7
Knee issues	291	15.3	24.0
Calf strain	159	8.4	13.1
Achilles tendinitis or tear	131	6.9	10.8
Lower leg or ankle tendinitis not involving Achilles	111	5.8	9.2
Stress fracture involving tibia or fibula	23	1.2	1.9
Other lower leg injuries	18	0.9	1.5
Ankle sprain	131	6.9	10.8
Plantar fasciitis	129	6.8	10.6
Stress fracture involving foot	41	2.2	3.4
Morton's neuroma	38	2.0	3.1
Metatarsalgia	38	2.0	3.1
Great toe metatarsal phalangeal joint pain (bunion)	30	1.6	2.5
Other foot and ankle injuries	54	2.8	4.5
Skin wounds, blisters, and infections	18	0.9	1.5
Other not previously specified	18	0.9	1.5

A comparison of select characteristics of those reporting an exercise-related injury in the prior 12 months with those who had not been injured is shown in Table 6. Compared with the uninjured group, those who had suffered an injury during this time period were younger, less experienced runners, who had relatively less focus on running, spent a greater proportion of their exercise time at a high intensity, and were more likely to have performed regular resistance training. Analyzing men and women separately, body mass index (BMI) was not different between groups.

**Table 6:** Comparison of select characteristics of those reporting an exercise-related injury in the prior 12 months with those who had not been injured.

	With Injury	Without Injury	
Characteristic	n = 933	n = 279	*P*
Age (years)	42.3±10.5	44.7±10.9	0.0008
Sex (% women)	32.3	31.2	0.77
Running experience (years)^a^	15±11	17±12	0.0060
Ultramarathon running experience (years)^b^	5±7	7±7	0.0008
Running distance in past year (km)	3,347±1,407	3,413±1,392	0.29
Lifetime running distance (km)	27,906±32,381	30,670±36,604	0.15
Average lifetime running distance per year (km)	2,197±2,771	1,950±1,541	0.18
Running on concrete or asphalt in past year (%)	42±27	45±28	0.15
Running on concrete or asphalt in lifetime (%)	55±25	55±26	0.85
Relative exercise time spent running in past year (%)	82±18	85±17	0.0017
Relative exercise time spent running in lifetime (%)	70±23	76±20	0.0001
Exercise time at high intensity in past year (%)^c^	24±19	23±18	0.50
Exercise time at high intensity in lifetime (%)^c^	23±18	20±15	0.0050
Performed resistance exercise in the past year (%)^d^	48.1	39.8	0.16
Performed resistance exercise in lifetime (%)^d^	72.9	64.9	0.11

Data are reported as mean ± SD except for group percentages.

^a^ Running experience was based upon the year the subject started running at least 3 days per week.

^b^ Ultramarathon running experience was based upon year first ultramarathon was run.

^c^ High intensity was defined as "will break a sweat after 3–5 minutes; breathing is deep and rapid; can only talk in short phrases."

^d^ An affirmative response meant the subject had regularly performed resistance training for at least a continuous 3-month period of time.

Select characteristics were also compared between those who had and had not reported an exercise-related stress fracture in the prior 12 months (Table 7). Compared with the group not having had a stress fracture, those sustaining a stress fracture during the prior year were younger and more likely to be a woman, were less experienced at ultramarathon running, had run a greater distance during the year, spent a greater proportion of their exercise time at high intensities, were less likely to have performed regular resistance training over the course of their lifetime, and were more likely to have had a prior history of exercise-related stress fracture. BMI did not differ between groups when considering men and women separately. Among the women, 40% reported that they had been taking calcium, vitamin D, or a prescription drug for bone health for at least the prior 2 years. The proportion taking these medications did not differ between those who had and had not sustained a stress fracture in the prior year.

**Table 7:** Comparison of select characteristics of those reporting an exercise-related stress fracture in the prior 12 months with those who had not suffered as stress fracture.

	With	Without	
	Stress Fracture	Stress Fracture	
Characteristic	n = 67	n = 1145	*P*
Age (years)	39.3±8.6	43.1±10.7	0.0070
Sex (% women)	49.3	31.0	0.0028
Running experience (years)^a^	13±10	16±12	0.060
Ultramarathon running experience (years)^b^	4±4	6±7	0.032
Running distance in past year (km)	3,876±1,526	3,332±1,390	0.0039
Lifetime running distance (km)	24,288±26,848	28,790±33,740	0.17
Average lifetime running distance per year (km)	2,259±1,918	2,133±2,576	0.76
Running on concrete or asphalt in past year (%)	41±26	43±27	0.53
Running on concrete or asphalt in lifetime (%)	51±27	55±25	0.23
Relative exercise time spent running in past year (%)	81±17	83±18	0.21
Relative exercise time spent running in lifetime (%)	69±24	72±22	0.33
Exercise time at high intensity in past year (%)^c^	31±25	24±19	0.064
Exercise time at high intensity in lifetime (%)^c^	27±19	22±17	0.033
Performed resistance exercise in the past year (%)^d^	44.8	46.3	0.23
Performed resistance exercise in lifetime (%)^d^	59.7	71.7	0.038
Prior history of exercise-related stress fracture (%)	47.8	20.8	<0.0001

Data are reported as mean ± SD except for group percentages.

^a^ Running experience was based upon the year the subject started running at least 3 days per week.

^b^ Ultramarathon running experience was based upon year first ultramarathon was run.

^c^ High intensity was defined as "will break a sweat after 3–5 minutes; breathing is deep and rapid; can only talk in short phrases."

^d^ An affirmative response meant the subject had regularly performed resistance training for at least a continuous 3-month period of time.

A prior lifetime history of an exercise-related stress fracture was reported by 302 (24.9%) of the group. Nearly half (45.0%) of the individuals with a stress fracture history indicated one had been sustained after they had started ultramarathon running, and most (88.1%) indicated one had been sustained after they had started running at least 3 days per week. As shown in Table 8, the most common sites were the foot (44.5%) and lower leg and ankle (42.7%) with prevalences of 12.4% and 11.9%, respectively.

**Table 8:** Number of active ultramarathon runners (among 1,212) reporting a lifetime history of a stress fracture involving specified body sites and relative distribution and prevalence of stress fracture for each site.

Body Site	n	Distribution (%)	Prevalence (%)
Foot	150	44.5	12.4
Lower leg/ankle	144	42.7	11.9
Leg/hip	30	8.9	2.5
Pelvis	8	2.4	0.7
Other	5	1.5	0.4

Note that some individuals reported multiple episodes of stress fractures at a given site which is not accounted for in these data.

Study participants were asked if they had ever required hospitalization after a competitive event. Fifty-nine individuals (4.9%) reported 63 hospitalizations after a competitive event. Of those, five hospitalizations were reported to have been associated with events other than running. Excluding those five hospitalizations, the primary causes were reported as dehydration, electrolyte disturbance or heat exhaustion (53.3%); fracture or dislocation (20.0%); skin injuries, including blisters and wounds (5%); concussions (5%); soft tissue injuries (5%); and infections (5%). Among those who reported dehydration, electrolyte disturbance, or heat exhaustion as the cause for hospitalization, 19.4% specified that hyponatremia was involved.

## Discussion

It might be presumed that individuals who are capable of running ultramarathons are healthier and have fewer medical needs than a comparable general population, except perhaps for those issues related to injuries from their exercise. The present study generally supports this premise. Compared with self-reported data from the general population, the prevalence of virtually all chronic diseases and mental health disorders appeared lower in the ultramarathon runners [15]–[17]. Asthma and allergies/hay fever were notable exceptions: The prevalence of these chronic conditions was higher among the ultramarathon runners than the general population. The respective prevalence for asthma and allergies was around 11% and 25% in the ultramarathoners, whereas the prevalence in American adults is around 8% and 7%, respectively [17]. Although physical activity may be a protective factor against asthma development [18], asthma is more common among endurance athletes, most likely the result of drying of the airways during exercise [19]. Additionally, the prevalence of allergies in recreational marathon runners was recently shown to be higher than in the general population, possibly due to greater exposure to airborne allergens [20]. As such, the finding of higher incidence of asthma and allergy among ultramarathon runners compared with the general population is not surprising.

**Figure 1:**
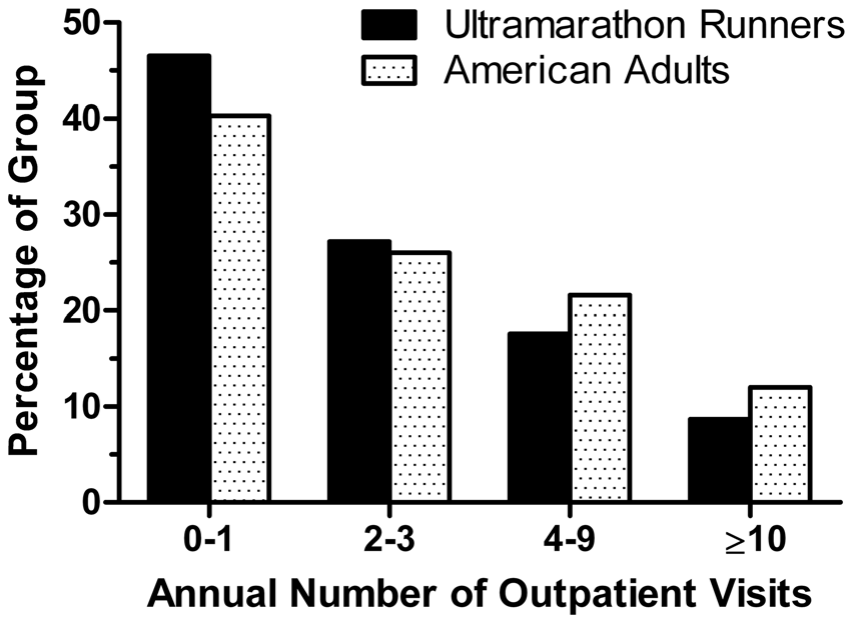
Comparison of annual rates of outpatient medical visits between ultramarathon runners and adult Americans. The percentage of individuals with specified number of annual outpatient medical visits, excluding dental and emergency room visits, are displayed. Adult American data are for those 18 through 64 years of age for the year 2011 as reported by the U.S. Department of Health and Human Services [17]. The Chi-square test showed a significant (p<0.0001) group difference.

The present study found that the ultramarathoners had missed an average of 2.2 days of work or school in the previous 12 months due to injury or illness. This is only about 60% of the 3.7 day average reported for employed Americans aged 18 through 74 years in 2011 [17]. Furthermore, the average number of days in which more than half the day was spent in bed due to any illness or injury in the prior year was 1.0 among the ultramarathoners, compared with the 4.7 days reported for adult Americans [17]. These findings support our previous research indicating that ultramarathon runners lose fewer work or school days due to illness or injury than the general population [12]. This is partially explained by the fact that ultramarathon runners tend to have higher levels of education [12], [14], [21] and are more likely to have white-collar occupations than the general population [12], [14], [21], [22] making it less likely that minor medical issues prevent performance of work duties. Indeed, those with higher education and income have less work loss and days in bed than lesser educated and poorer individuals [17]. Even when compared with the highest education category (bachelor's degree or higher) and the group with the highest annual family income ($100,000 or more), the ultramarathon runners still reported less work loss and fewer days in bed due to injury or illness. Ultramarathon runners were also found to have a lower frequency of outpatient medical visits than the general population. Figure 1 shows a comparison of the study group of ultramarathon runners with American adults of comparable age [17].

It is important to note that the ultramarathoners only lost an average of 0.3 days from work or school in the prior 12 months due to an exercise-related injury. Although exercise-related injuries were quite common among this group (64.6% had experienced an injury in the prior 12 months that resulted in a median of 14 days lost training), these injuries did not, in most cases, lead to missed work or school. Also of note was that medical visits specific to an exercise-related issue accounted for over 60% of the use of the medical care system by the ultramarathoners.

The annual injury incidence rate among long-distance runners has previously been reported to be around 50–60% [23]–[26], and our prior study of a large cohort of 161-km ultramarathon entrants found that 52% had suffered injuries severe enough to interfere with training in the previous year [5]. Recognizing variations among studies in the way an injury has been defined, it appears that the injury incidence rate in the present study population is similar to what has been previously reported. This is of interest since running distance has been found to be a risk factor for injury [27], and, with the exception of the 161-km ultramarathon entries we examined [5], the present group averaged greater running distances than the runners in the other studies. Clearly, there are determinants of injury besides distance run. This is evident from the data from the current study indicating that average annual running distances were similar for those who had and had not sustained an injury during the year (Table 6). Perhaps those who have withstood the stress associated with ultramarathon running have better adapted for such demands and/or have favorable intrinsic characteristics that reduce their injury risk relative to those who are running shorter distances. The presumed greater use of running surfaces other than concrete or asphalt may also offer some protection from certain injuries. Differences in relative intensity and running speed, along with the associated effects this might have on stride frequency, foot strike pattern and impact forces, could also be of importance in limiting injuries among ultramarathon runners [28].

The active ultramarathon runners who had sustained an exercise-related injury in the prior year were distinguished by several characteristics from those who remained free of injury. Those sustaining an injury were younger and less experienced at running, had relatively less focus on running, spent a greater proportion of their exercise time at high intensities, and were more likely to have performed regular resistance training. In comparing those who had and had not suffered an exercise-related stress fracture in the prior year, the results were similar as for all exercise-related injuries except that those with stress fractures were more likely to be women, had run a greater distance in the past year, and were less likely to have performed regular resistance exercise. Those individuals who reported a stress fracture in the prior year were also more likely to have had a prior history of exercise-related stress fracture.

Evidence is conflicting regarding age as a risk factor for running injuries, and there is limited support that being a woman and inexperienced at running are risk factors for running injuries [27]. The present results support prior findings based on studies of shorter distance runners that running inexperience may be a risk factor for injury. However, although being a woman was associated with greater risk of a stress fracture, it was not associated with a greater overall injury risk among the ultramarathon runners. Additionally, the present study found that greater age is associated with a lower injury risk among ultramarathon runners. This apparent reduction in injury risk associated with aging and greater running experience may simply reflect the adaptations resulting from years of running and/or from running on irregular terrain, enhanced knowledge from experience, and/or the presence of favorable intrinsic characteristics that have allowed the individual to continue participating in ultramarathon running.

The most common site of self-reported injury among the present study population was the knee, which accounted for 15% of the injuries. The knee has been shown to be the most vulnerable site for injury in prior reports of distance runners [27]. Our previous study also showed the knee to be the most common site of injury among 161-km ultramarathoners, accounting for 20% of all injuries [5]. In the ultramarathon runners, stress fractures accounted for 3.7% of exercise-related injuries, and 67 (5.5%) reported having suffered a stress fracture in the prior year. It has previously been reported that stress fractures account for 5–16% of all injuries in runners [29]–[32], and annual incidence has been reported to be as high as 25.9% among a group of college middle and long distance runners [33]. It has also been a typical finding that over half of the stress fractures in runners involve the lower leg [31], [33], [34]. Yet, our prior study of 161-km ultramarathon runners was more consistent with the present findings in that the site accounting for the highest percentage of stress fractures was the foot at 48% [5]. Therefore, it appears that stress fractures may be a relatively less common issue in ultramarathon runners compared with shorter distance runners, but are relatively more common in the foot among ultramarathon runners than shorter distance runners. We presume that the lower incidence of stress fractures in ultramarathon runners relates to the fact that only around half of their running is on concrete or asphalt (Tables 6 and 7), but that the higher distribution of stress fractures involving the foot is due to greater demands on that structure from running on irregular terrain.

The reported history of hospitalization after a competitive event by less than 5% of the group indicates that the need for such medical intervention is not especially common among ultramarathon runners when considering the number of years of exposure involved. On the other hand, it underscores that participation in the activity is not completely without potential serious medical consequences. Self-reported diagnoses of dehydration, electrolyte disturbance, or heat exhaustion accounted for over half of the hospitalizations, although the legitimate concern and need for hospitalization for clinically severe dehydration and heat illness has been questioned [6]. Interestingly, roughly one in five of these hospitalizations were indicated to be for hyponatremia. The incidence of hyponatremia at 161-km ultramarathons in northern California has been found to range from 5% to 51% [35], but the reports of hospitalization for hyponatremia in ultramarathons have been limited [36], [37] and seem to be more typically, at least in some events, when there is associated rhabdomyolysis [3], [37]. Proper education of runners about prevention of exercise-associated hyponatremia and of event medical personnel about its recognition and management should reduce the need for hospitalization for hyponatremia. In contrast, fracture or dislocation injuries, which accounted for 20.0% of hospitalizations, are not likely to be avoidable given that falls cannot be eliminated.

This investigation has the limitations imposed by self-selection recruitment methods, data collection by self-report, and reliance on published general population data for comparison rather than a control group. Self-selection bias does not appear to be a serious issue since many characteristics of this sample, some of which are reported elsewhere [13], [14], are comparable to prior reports of ultramarathoners [2], [5], [12]. On the other hand, the extent of self-report and recall bias cannot be quantified and may have affected the results, particularly relative to the data on lifetime exercise habits. The data were examined for outliers, and though it is possible that some incorrect entries could not be identified and corrected, the general conclusions of the study appear robust and well supported.

The present work provides an analysis of medical issues in a large cohort of ultramarathon runners. As expected, the work demonstrates that, with the exception of asthma and allergies, ultramarathon runners have fewer chronic medical conditions than the general population, tend to miss little time from work or school due to illness or injury, and make limited use of the medical care system. However, they have a small prevalence of serious medical issues that should be recognized by those providing medical care at ultramarathon running events. Ultramarathon runners also have a high annual incidence of exercise-related injuries that appears comparable to that of shorter distance runners. The most common site of injury for ultramarathoners is the knee, as is the case for shorter distance runners, but stress fractures are more common in the foot among ultramarathoners compared with shorter distance runners. Those ultramarathoners who sustain exercise-related injuries tend to be younger and less experienced than those who avoid injury.
